# Impact of sodium-glucose cotransporter-2 inhibitor use on peak VO_2_ in advanced heart failure patients

**DOI:** 10.3389/fcvm.2024.1376645

**Published:** 2024-07-24

**Authors:** A. Desai, S. Sharma, N. Abuah, J. Jang, S. Desai, S. Paghdhar, R. M. Goswami

**Affiliations:** Division of Advanced Heart Failure and Transplant Cardiology, Mayo Clinic, Jacksonville, FL, United States

**Keywords:** heart failure, diabetes, SGLT2i, exercise test, CPET, functional capacity, transplant, VO_2_

## Abstract

**Introduction:**

Advanced heart failure (HF) is an epidemic that affects multiple organ systems with high morbidity and mortality rates despite optimal medical therapy (OMT) and remains the leading cause of hospitalizations in type 2 diabetes-related cardiovascular disease. The addition of sodium-glucose co-transporter inhibitors (SGLT2i) in treating these patients has seen improved mortality and hospital admission rates. As such, we felt it was important to investigate whether the use of SGLT2i improved functional capacity in patients with HF when compared to OMT by evaluating maximum oxygen consumption (peak VO_2_) using cardiopulmonary exercise testing (CPET).

**Methods:**

We found 94 heart failure patients between August 2020 and August 2021 who underwent CPET before and after treatment at Mayo Clinic in Florida. 50 patients received OMT and 44 received OMT and SGLT2i therapy. CPET results before and after were compared for each group.

**Results:**

The baseline ejection fraction was not significantly different between groups, with the OMT group at 38% and the SGLT2i group at 33%, *p* = 0.10. OMT patients were found to have a significantly lower hemoglobin A1c of 5.7 (5.4–6.1) compared to those with SGLT2i therapy of 6.4 (5.8–7.1), *p* = 0.01. The baseline peak VO_2_ was 17.3 ml/kg/min (13.3–21.6) in the OMT group and 17.3 ml/kg/min (14.4–18.9) in the SGLT2i group, *p* = 0.18, not significantly different. The interesting finding is that the follow-up peak VO_2_ at one year for the OMT group was 17 ml/kg/min (13.3–21.6), which was not significantly different from the SGLT2i group peak VO_2_ of 17 ml/kg/min (14.6–19.6), *p* = 0.19. Our study is the first to compare before and after peak VO_2_ values of the OMT+SGLT2i group to the patient's own baseline and we found no significant improvement.

**Conclusion:**

Our single-center data shows no improvement in functional capacity after the addition of SGLT2i therapy to OMT in patients with advanced heart failure. Improved hospitalization and symptoms may be attributed to other numerous effects of SGLT2i such as volume management.

## 1 Introduction

Advanced heart failure (HF) is a multifaceted disease process that affects multiple organ systems. Often, patients with HF have concomitant glucose intolerance manifested as diabetes mellitus in conjunction with chronic renal insufficiency, associated with their diabetes and underlying renal disease processes. Compared to non-diabetic patients, those with type 2 diabetes (T2D) have an increased risk of developing HF. HF is the leading cause of hospitalizations in T2D-related cardiovascular disease (1). There remains an increased risk for morbidity and mortality in patients with HF despite the use of optimal medical therapy (OMT) such as beta-blockade, renin angiotensin aldosterone system (RAAS) inhibition, angiotensin-converting enzyme inhibitors, neprilysin inhibitors or angiotensin receptor blockers (2).

Outcomes with the addition of sodium-glucose cotransporter 2 inhibitors (SGLT2i) in the management of both systolic and diastolic HF patients have demonstrated decreased cardiovascular mortality and readmission rate (3). We felt it important to understand the effect on patients with advanced HF with or without T2D and determine if there was a change in functional capacity after SGLT2i initiation compared to those on OMT alone.

In order to create a streamlined understanding of the testing we evaluated, we provide a detailed background below that highlights the role of cardiopulmonary function testing and SGLT2i in the advanced heart failure population.

### 1.1 Cardiopulmonary exercise testing and exercise intolerance in advanced heart failure

Cardiopulmonary exercise testing (CPET) has become an established method for diagnosing cardiopulmonary diseases and their severity, providing prognostic information, gauging response to clinical therapy, and serving as a potential tool for assessing early states of disease and better identify and optimize therapeutic interventions. CPET establishes maximum exercise capacity by calculating the peak oxygen uptake at maximal exercise (peak VO_2_). It's the gold standard for objective measurement of exercise intolerance in HF patients (4). Cutoffs for peak VO_2_ in the HF population were defined by Mancini et al. in their seminal work, which has aided in its use in determining survival, and response to therapy, with its integration as part of the international society of heart and lung transplant (ISHLT) guidelines for the assessment of advanced therapy in HF patients (5).

**Figure 1:**
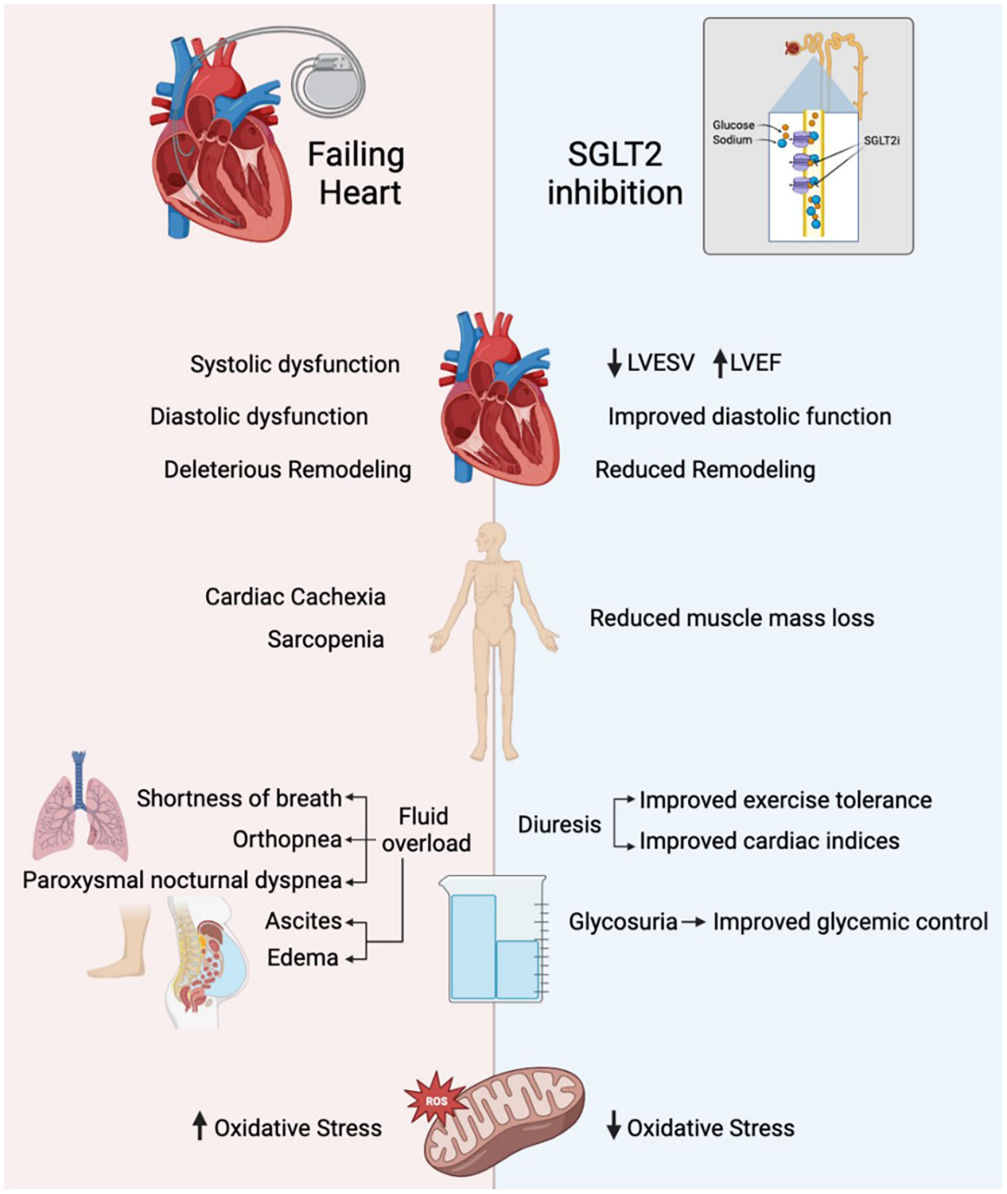
NSGLT2i and their role in exercise tolerance. LVESV, left ventricular end systolic volume; LVEF, left ventricular ejection fraction.

Varying degrees of exercise intolerance is always present in patients with HF and is due to numerous underlying mechanisms such as fibroblast-induced remodeling of the myocardium, systolic and diastolic dysfunction, altered calcium handling and increased oxidative stress (Figure 1) (4). In addition, conditions such as fatigue, dyspnea, anorexia, sarcopenia, and anemia are associated with cardiac cachexia, which contributes to poor physical performance and outcomes. Cardiac cachexia involves the dreadful loss of edema-free muscle mass and may be accompanied by fat mass depletion (6). It affects 10%–22% of all heart failure patients (7). Sarcopenia is another such consequence that refers to a decline in skeletal muscle mass through atrophy and cellular remodeling that is usually associated with aging but accelerated by comorbidities has a prevalence of 34% in patients of HF (8, 9).

### 1.2 Sodium-glucose-like transporter type 2 inhibitors and exercise tolerance

Experimental and clinical research has shown that SGLT2 inhibitors affect exercise tolerance through a wide range of effects on cardiac myocytes, skeletal muscle, and on adipose tissue which could potentially counteract the mechanisms by which HF causes exercise intolerance (4). SGLT2i use can result in improved systolic and diastolic function, reduced cardiac remodeling, and reduced oxidative stress (Figure 1). The EMPA-Tropism study showed improvements in left ventricular ejection fraction (LVEF) and left ventricular end-systolic volumes (LVESV), which was consistent with the SUGAR-DM-HF trial. However, this is inconsistent throughout the literature, with limited LV remodeling after 3 months of empagliflozin and one year of dapagliflozin (10–13).

Overall, however, the class of SGLT2 inhibitors have demonstrated a reduction in oxidative stress, improved lipid oxidation, and weight loss, all of which contribute to enhanced exercise performance, potentially evidenced by a reduction in NT-Pro-BNP (4, 11).

### 1.3 Sodium-glucose-like transporter type 2 inhibitors and volume management

SGLT2 inhibition decreases sodium reabsorption in the proximal tubule and promotes sodium delivery to the macula densa. This leads to tubuloglomerular feedback (TGF) activation, subsequent afferent arteriole vasoconstriction, and reduced glomerular filtration rate. The SGLT2i mechanism ultimately involves decreasing proximal tubule reabsorption of glucose and sodium, thereby enhancing osmotic diuresis and glycosuria and improving glycemic control and a patient's intravascular volume status (14).

There seems to be a significant improvement in left ventricular filling pressure related to increased osmotic diuresis (15). Enhanced diuresis also aids symptom management in HF as many of the symptoms that arise, such as shortness of breath, dyspnea of exertion, weight gain, orthopnea and paroxysmal nocturnal dyspnea stem from HF-induced volume overload (16).

Building upon the role of SGLT2i and improvement in the circulating volume status, a known factor associated with acute HF decompensation, the DAPA-HF study demonstrated a reduction in cardiovascular death with dapagliflozin, and the EMPA-REG OUTCOME also showed a decrease in mortality with empagliflozin (17, 18). Interestingly as Shanmuganathan and colleagues discussed in their letter to the editor for the EMPEROR- Reduced, many trials related to the SGLT2i class fail to disclose if and any adjustment is needed in reducing diuretic dosing for HF patients, given the significant osmotic diuresis that is known to occur, to prevent an acute renal injury (19). It remains unknown if optimization of excess volume alone in patients with advanced HF, New York Heart Association (NYHA) Class 3–4, improves functional capacity in a way that may obviate the need for organ transplantation.

We discern that even though the data from major clinical trials shows a benefit to readmission and mortality with SGLT2i use, this does not necessarily highlight the long-term impact upon the advanced heart failure patient – specifically transplant or LVAD implant. Furthermore, SGLT2i therapy and OMT have not clearly demonstrated a marked improvement in physical performance in chronic heart failure, where the cardiac muscle has undergone deleterious remodeling (4). The focus on peak VO_2_, patient effort (RER), and VE/VCO_2_ may allow one to elucidate potential non-responders to SGLT2i therapy who may need to be aggressively risk-stratified, optimized, or considered for advanced therapies earlier in their management - making a potential impact on their survival.

While these benefits of SGLT2 inhibitors on the cardiovascular system and exercise physiology are well documented in those without HF, there is limited data in the advanced HF population. Given the life-altering effects of undergoing a workup for heart transplantation or a durable left ventricular assist device (LVAD), the role of exercise and cardiovascular remodeling in this population could be significant. Our study aims to explore the potential for SGLT2 inhibition to improve exercise tolerance and therefore the quality of life in patients with HF to an extent that may demonstrate significant improvements in standardized cardiopulmonary testing and potentially delay need for transplant or LVAD.

## 2 Methods

After IRB approval, we performed a retrospective review of patient records between August 2020 and August 2021. All patient data was extracted from the electronic medical record. We included all patients with a confirmed diagnosis by ICD-10 code of either systolic (I50.2), diastolic (I50.3), or combined systolic and diastolic heart failure (I50.42). We then verified that a CPET result was available at baseline and one year after either the initiation of SGLT2i or maximally tolerated OMT. Our institution's advanced heart failure team judged OMT tolerance based on provider documentation in the electronic medical record. 13,213 patients were found to be on SGLT2i during our review period. Of these, 2,063 fulfilled one of the three ICD-10 codes. Out of these, 94 patients were identified with a baseline and follow-up CPET within one year of optimization of medical therapy or initiation of SGLT2i therapy.

Our control group, the OMT group, were the patients found to be taking guideline directed OMT (20). Our study group, OMT+SGLT2i group, had an additional SGLT2i (dapagliflozin) in their regimen. Of the 94 patients with CPET results available, 50 patients with OMT while 44 had OMT with the addition of an SGLT2i (dapagliflozin).

The primary endpoint was the effect of SGLT2i on Peak VO_2_ recorded during CPET. We also report outcomes in terms of progression of therapy to LVAD/transplant and mortality. We report demographics, baseline medical therapies, follow-up duration, and relevant patient-specific parameters such as body mass index, renal function, and HbA1c levels.

We report median values and interquartile range for these parameters. Where appropriate, descriptive, and comparative statistical analyses were performed using the Mann-Whitney or chi-square analysis to determine statistical significance. IBM SPSS version 27 was used for statistical analysis. *P* value of ≤0.05 was considered statistically significant for all outcomes.

### 2.1 Outlining optimal medical therapy

Patients were on optimal medical therapy based on definitions of target doses for medications outlined within the 2021 American College of Cardiology (ACC) consensus statement (20). For those individuals within the study population before 2021, SGLT2i may not have been an option; as such, many patients within our OMT cohort were optimized on all other therapies outlined within the 2021 ACC consensus statement – as tolerated by symptoms or medication side effects requiring dose reduction or cessation of treatment at the discretion of the treating provider.

### 2.2 Standardization of CPET

**Figure 2:**
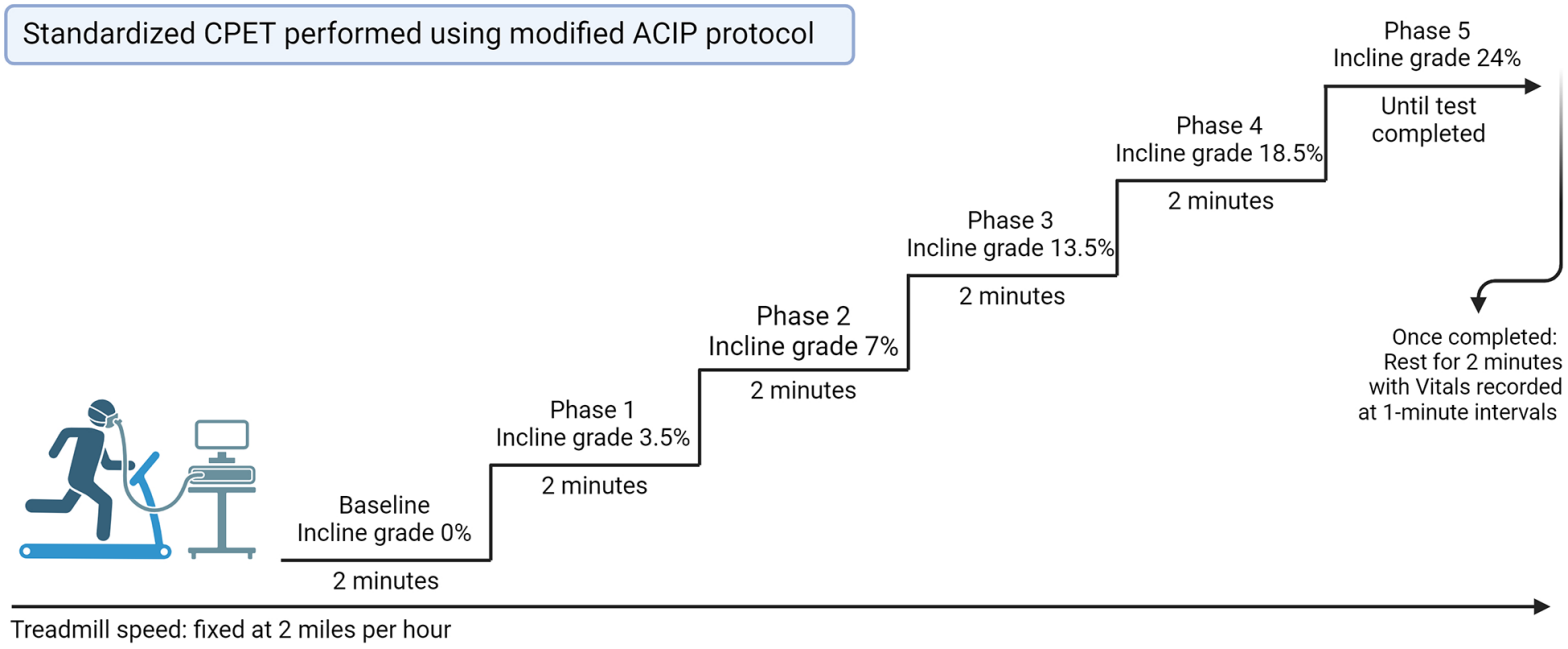
Standard CPET performed using modified ACIP protocol. CPET, cardiopulmonary exercise test; ACIP, Asymptomatic Cardiac Ischemia Pilot.

Our institutional standard for CPET was utilized on all patients with HF being seen in the advanced HF outpatient setting (21). CPET was performed using the modified Asymptomatic Cardiac Ischemia Pilot (ACIP) protocol which is as follows: baseline pretest measurement recording of breathing to calibrate the machine, blood pressure and heart rate. Exercise is initiated at a fixed rate of 2 miles per hour treadmill speed, with an incline at 0%. At 2 min the patient incline is increased to 3.5% grade, at 3 min it is increased to 7% for 2 min, then to 13.5% for 2 min, followed by 18.5% for 2 min followed by 24% until test completion (Figure 2). Post-procedure, the patient has a 2-min rest period with vitals recorded at one-minute intervals. All data from CPET testing were only interpreted by advanced heart failure cardiologists at the time of testing, and an independent provider reviewed all CPET data for this study for consistency.

## 3 Results

94 patients were identified with a baseline and follow-up CPET within one year of optimization of medical therapy or initiation of SGLT2i therapy. 50 patients undergoing OMT while 44 had OMT with the addition of an SGLT2i (dapagliflozin). Baseline patient characteristics, relevant parameters and outcomes are outlined in Table 1. CPET results are reported in Table 2.

**Table 1:** Baseline patient characteristics.

	OMT group (*n* = 50)	OMT+SGLT2i group (*n* = 44)	*P*-value
Demographics			
Age (median, IQR)	62 (52–70)	59 (53–64)	0.08
Female (#, %)	16 (32%)	17 (38%)	0.31
HF classification and ejection fraction (median, IQR)			
Ejection fraction (%)	38 (25–50)	33 (25–40)	0.10
HFrEF (%)	25 (21–34)	27 (22–33)	0.41
HFpEF (%)	53 (45–57)	50 (40–60)	0.30
Etiology (#, %)			
Ischemic	13 (26%)	19 (43%)	0.06
Non-ischemic	37 (74%)	25 (57%)	
Medical therapy (#, %)			
Beta-blocker	41 (82%)	40 (91%)	0.09
ARNI	39 (78%)	28 (63%)	**0** **.** **05**
ACEi	1 (2%)	6 (13%)	**0**.**02**
ARB	4 (8%)	5 (12%)	0.22
MRA	30 (68%)	31 (70%)	0.16
Hydralazine	1 (2%)	1 (2%)	1.0
Hemoglobin A1c (%) (median, IQR)	5.7 (5.4–6.1)	6.4 (5.8–7.1)	**0**.**01**
Diabetics (#, %)	0 (0%)	20 (45%)	–
Body Mass Index (kg/m^2^) (median, IQR)	28.5 (25.5–31.4)	30.9 (27.4–30.8)	**0**.**008**
Estimated glomerular filtration rate (ml/min/1.73 m^2^) (median, IQR)			
Baseline	70 (57–84)	67 (60–84)	0.39
At one year follow up	63 (51–75)	67 (55–78)	0.25
Patient outcomes			
LVAD/transplanted (#, %)	12 (24%)	8 (18%)	–
Deceased (#, %)	3 (6%)	1 (2%)	–
Days between CPET (median, IQR)	352 (298–460)	437 (291–569)	0.07
CPET results (median, IQR)			
Baseline peak VO_2_ (ml/kg/min)	17.3 (14.3–22.3)	17.3 (14.4–18.9)	0.18
Peak VO_2_ at one year (ml/kg/min)	17 (13.3–21.6)	17 (14.6–19.6)	0.19
Difference from baseline (ml/kg/min)	−0.3 (−1.6 to 0.95)	0.6 (−1.7 to 2.0)	0.48

CPET, cardiopulmonary exercise test; HF, heart failure; HFrEF, heart failure with reduced ejection fraction; HFpEF, heart failure with preserved ejection fraction.

Bold *p* values are statistically significant at *p* < 0.05.

**Table 2:** Cardiopulmonary exercise test parameters.

Parameters (median, IQR)	OMT group (*n* = 50)				OMT + SGLT2i group (*n* = 44)			
	Baseline	1-year follow up	*P* value	Mean difference (95% CI)	Baseline	1-year follow up	*P* value	Mean difference (95% CI)
Exercise time (minutes)	7.2 (6–9.1)	7.6 (5.7–9.5)	0.48	0.1 (−0.5 to 0.6)	7.2 (5.6–8.0)	7.15 (5.5–8.4)	0.44	−0.1 (−0.6 to 0.4)
Resting HR (bpm)	60 (61–80)	69 (62–77)	0.24	1.6 (−1.7 to 4.9)	74 (66–79)	72 (67–79)	0.42	0.9 (−2.6 to 4.4)
Peak HR (bpm)	127 (114–142)	120 (105–144)	0.20	4.38 (0.1–8.7)	125 (114–137)	121 (107–134)	0.29	3.1 (−1.5 to 7.7)
Peak HR % predicted (%)	77.5 (69–85.5)	75.5 (65.2–85.2)	0.27	1.9 (−0.7 to 4.5)	75.0 (67.5–81.5)	74.5 (67–80)	0.45	0.04 (−4.1 to 4.2)
Peak systolic pressure (mmHg)	124 (106–154)	122 (110–144)	0.47	−0.64 (−7.4 to 6.2)	134 (120–151)	133 (122–148)	0.22	−6.9 (−16.9 to 3.17)
Peak RER	1.2 (1.1–1.3)	1.2 (1.1–1.3)	0.46	−0.002 (0.04–0.03)	1.2 (1.1–1.2)	1.2 (1.1–1.2)	0.40	0.01 (−0.02 to 0.05)
Peak METS, measured (METS)	5.0 (4–6.3)	4.8 (3.8–6.3)	0.46	0.1 (−0.1 to 0.3)	4.9 (4.1–5.2)	4.9 (4.0–5.6)	0.42	0.07 (−0.3 to 0.4)
O2 pulse (ml)	11.8 (9.8–16)	12.1 (10.0–15.5)	0.41	−0.1 (−0.7 to 0.4)	13.0 (10.3–16.4)	13.1 (10.6–15.9)	0.43	−0.3 (−1.5 to 0.8)
VE/VCO2 slope	32.5 (30.0–38.0)	34.2 (30.8–36.5)	0.35	−0.7 (−2.3 to 0.9)	32.0 (28.0–35.0)	32.2 (29.0–34.8)	0.42	0.3 (−0.9 to 1.6)
Peak speed (mph)	3 (2–3)	2.4 (2–3)	0.43	−0.008 (−0.01 to 0.1)	2 (2–3)	2 (2–3)	0.44	0.009 (−0.14 to 0.16)
Peak incline (%)	14 (12.5–17.5)	14 (12.5–17.5)	0.35	0.5 (−0.6 to 1.6)	14 (12.5–16.5)	14 (12.5–14.0)	0.42	−0.08(−0.8 to 0.6)
peak VO_2_ (ml/kg/min)	17.3 (14.3–22.3)	17 (13.3–21.6)	0.39	0.41 (−0.38 to 1.21)	17.3 (14.4–18.9)	17 (14.6–19.6)	0.32	0.36 (−0.78 to 1.51)

RER, respiratory exchange ratio (VCO2/VO2).

### 3.1 Demographics

Within the OMT group, 32% were female compared to 38% in the OMT+SGLT2i group. The median age in the OMT group was 62 (52–70) years compared to 59 (53–64) in the OMT+SGLT2i group.

### 3.2 Cardiovascular, renal and metabolic parameters

Baseline ejection fraction (LVEF) was not significantly different between groups, 38% (IQR 25–50) in the OMT group and 33% (25%–40%) in the OMT+SGLT2i group, *p* = 0.10. There were more non-ischemic patients within the OMT group compared to the OMT+SGLT2i group, and this trended toward, but did not reach significance, *p* = 0.06. The median BMI of the OMT group was 28.5 kg/m^2^ (25.5–31.4) and of the SGLT2i group was 30.9 (27.4–30.8), *p* = 0.008.

Baseline medical therapy between the two groups showed similar profiles of beta-blocker (*p* = 0.09) and mineralocorticoid receptor antagonist (*p* = 0.16) use. More patients in the OMT group were on therapy with angiotensin-neprilysin inhibitors (39 vs. 28, *p* = 0.05) and more patients from the OMT+SGLT2i group were on ACEi therapy (6 vs. 1, *p* = 0.02). When all medications that affect the RAAS were analyzed as one group, there was a statistical difference (*p* = 0.03).

OMT patients were found to have a significantly lower hemoglobin A1c of 5.7 (5.4–6.1) compared to those with OMT+SGLT2i therapy of 6.4 (5.8–7.1), *p* = 0.01. Baseline and 1-year follow-up renal function were not different between the two groups, *p* = 0.25.

### 3.3 CPET data

The median duration between baseline and follow-up CPET was 352 days (298–460) in the OMT group and 437 days (291–569) within the OMT+SGLT2i group, *p* = 0.07.

Baseline peak VO_2_ was 17.3 ml/kg/min (13.3–21.6) in the OMT group and 17.3 ml/kg/min (14.4–18.9) in the OMT+SGLT2i group, *p* = 0.18. Follow-up peak VO_2_ at one year for the OMT group was 17 ml/kg/min (13.3–21.6), which was not significantly different than the OMT+SGLT2i group's peak VO_2_ of 17 ml/kg/min (14.6–19.6), *p* = 0.19.

**Figure 3:**
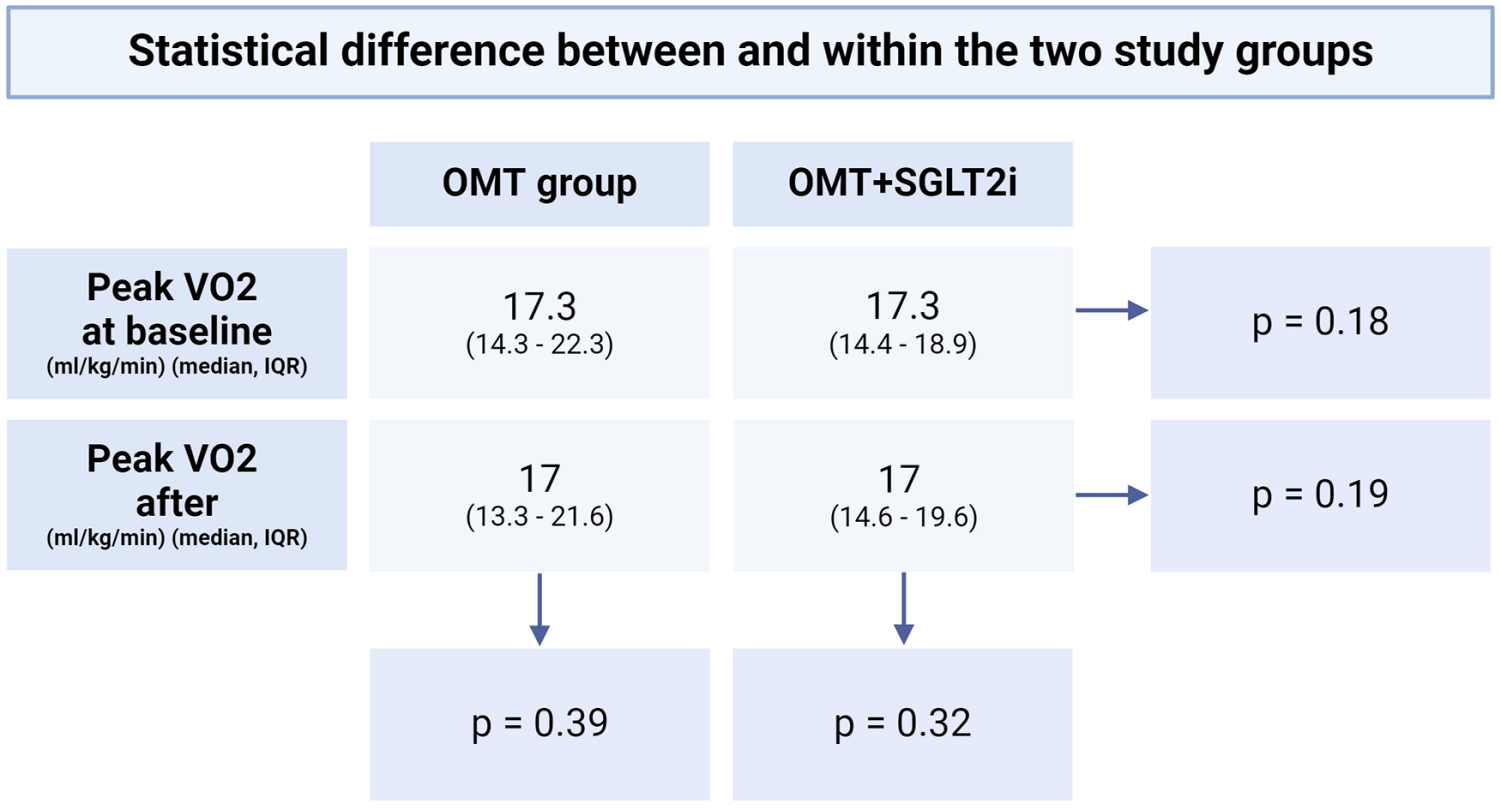
Statistical difference between and within the two study groups.

**Figure 4:**
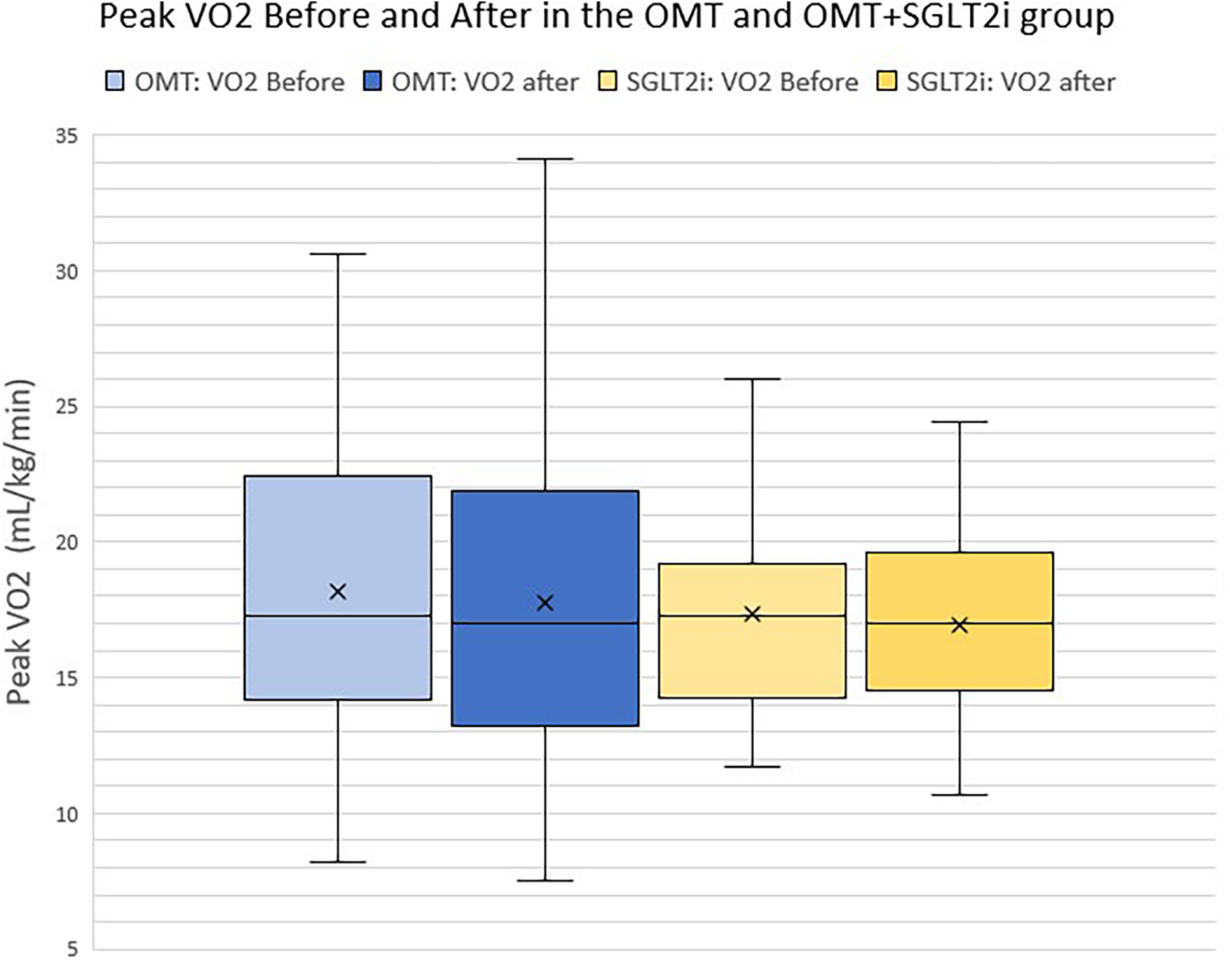
Peak VO2 before and after in the OMT and the OMG+SGLT2i group.

**Figure 5:**
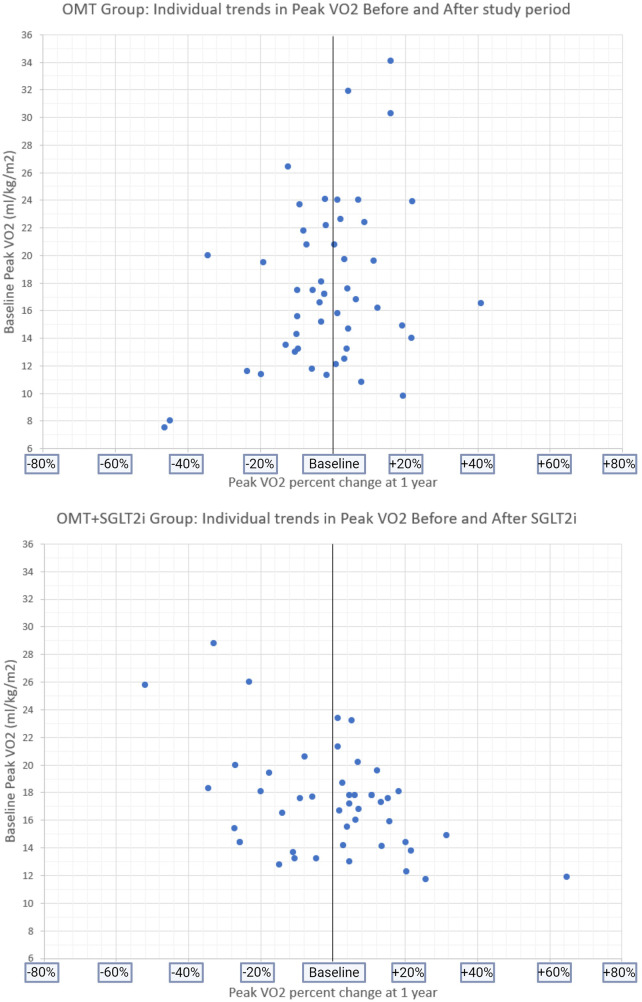
Baseline peak VO2 and individual PERCENT CHANGE in peak VO2 at follow-up in both study groups.

Based on literature review, we are the first to be able to compare a patient's own baseline peak VO_2_ before and after SGLT2i initiation (the OMT+SGLT2i group). The peak VO_2_ before initiating therapy for the SGLT2i group was 17.3 ml/kg/min (14.4–18.9) that did not increase significantly after adding an SGLT2i to OMT, with a 1 year follow up peak VO_2_ of 17 ml/kg/min (14.6–19.6), *p* = 0.32 (Figures 3–5).

In addition to peak VO2, various CPET parameters are outlined in Table 2 with statistical analysis. In both the OMT group and the OMT+SGLT2i group, there is no statistically significant difference in exercise duration between baseline and follow-up (*p* = 0.48 for OMT group; *p* = 0.44 for OMT+SGLT2i group). Similarly, the VE/VCO2 slope at baseline and follow-up does not exhibit any statistical difference in either group (*p* = 0.35 for OMT group; *p* = 0.42 for OMT+SGLT2i group). Peak METS in both groups also do not show any statistical difference (*p* = 0.46 for OMT group; *p* = 0.42 for OMT+SGLT2i group).

## 4 Discussion

This is one of the first manuscripts to propose that OMT+SGLT2i use alone does not improve functional capacity in advanced heart failure patients (NYHA 3+) as measured by peak VO_2_ during CPET. In this retrospective study, we evaluated all patients with heart failure (preserved or reduced ejection fraction) undergoing serial CPET over 12 months. After initiation of SGLT2i or while on maximally tolerated Optimal Medical Therapy (OMT), pre-CPET and post-CPET were compared within each group as well (Figure 3). For those patients receiving OMT alone 12(24%) patients received heart transplantation or durable left ventricular assist device (LVAD), and 3(6%) died. In the OMT+SGLT2i group, 8(18%) patients underwent heart transplantation or durable LVAD placement and 1(3%) died.

As we begin to understand the effects of novel therapies crossing disease borders and impacting the entire patient, we have learned that the pluripotent effects of medications in the modern era have unforeseen benefits in some cases. Reduction of the heart failure burden in the context of readmissions and improvement in functional capacity is important, however, to better understand the trajectory of heart failure and therapies such as transplant or LVAD, we need to better define risk categories for those patients with the highest risk, but potentially the greatest reward. As we look to traditional literature from Mancini, Packer, Weber, and others who created the platforms for modern medical optimization, it is imperative to not only place patients in the right risk group but work to optimize the individual potential of each medicine (3, 5).

As a result of such innovation, we present our data from a large transplant and advanced heart failure center that helps answer the next question, beyond physical improvements and readmissions, but that of true transformation of patient outcomes in those with advanced HF.

In our reviewed literature search, the current major clinical trials studying the effects of SGLT2i on heart failure patients fail to include the utilization or assessment of CPET and peak VO_2_ within the framework of their outcomes, with most favoring the 6-minute walk (6MW). For trials such as EMPA-REG, EMPEROR Preserved, CANVAS and DAPA-HF, CPET testing was not available (17, 18, 22, 23). For papers like EMPA-Tropism (2020), for example, CPET was considered novel (10). However, this was a placebo-controlled study and therefore did not compare each participant to OMT or to their own baseline. The suggestion proposed based on this trial is that the SGLT2i improves the functional testing, however, this clear correlation cannot be taken at face value based on their data. Volume optimization, an effect well understood due to osmotic diuresis in SGLT2i and the anti-inflammatory effect of less myocardial stress, is known to benefit myocardial contraction. As such, optimization of patients with salt restriction, fluid and diuretic therapy can also conceivably show similar results. In fact, He and colleagues in 2021 showed that aggressive diuresis can augment the response of patients with both HF reduced or preserved ejection fraction (24). Although the study was terminated prematurely due to new guideline recommendations regarding the use of SGLT2 inhibitors, the CANA-HF study (2020) reported similar findings to ours in a 12-week study that canagliflozin did not improve the peak VO_2_ and VE/VCO_2_ slope (25).

The latest 2021 ACC guidelines for the treatment of advanced heart failure saw the addition of new medications representing a significant advancement in the field, particularly SGLT2i and ARNI (20). Currently, the only approved ARNI in use is sacubitril/valsartan (S/A). ARNI and other medications affecting the RAAS system demonstrate favorable effects in the pathophysiology and symptomatology of heart failure and could potentially lead to improvements in peak VO_2_ (26, 27). In a prospective study with 37 patients, Cacciatore et al. showed increased VO2 max and improved 6MWT at 12 months follow up and compared to their baseline (28). Meanwhile, the PARALLAX trial showed no significant impact on exercise tolerance as shown by no improvement in the 6MW test at 24 weeks with ARNI use (29). Santos et al. used 6MW test and CPET to assess functional outcomes with ARNI therapy. The study reported no significant improvement in in 6MW or the peak VO_2_ when compared to their enalapril control group (30).

While parameters such as BMI may affect the peak VO_2_, studies have shown inconsistent results. Some studies show a negative correlation between BMI and peak VO_2_, attributing to the to the increase in type 2 muscle fibers and a decrease in type 1 muscle fibers in obese individuals and increased fat mass which may reduce oxygen uptake (31). Other studies demonstrate that BMI or fat percentage do not affect the peak VO_2_ but rather the exercise capacity of individuals (32). This may also be attributed to the lack of BMI to differentiate between fat and muscle mass. Diabetes is another such comorbidity known to affect CPET performance, although the evidence is inconclusive. One study found no significant difference in peak VO2 in diabetics when compared to well matched control group without diabetes (33). While the OMT+SGLT2i group in our study shows higher HbA1c levels and is comprised of 45% diabetics, there was no difference by true value (raw VO2) or in assessing a statistical difference in the baseline peak VO2 in the OMT or the OMT+SGLT2i group.

An observation worth noting is that in our population, we found a trend that showed a patient with higher baseline peak VO_2_ may have a significant drop but remain above the cutoff for advanced HF evaluation. This canary in the coal mine perhaps can, with larger data and multi-center support, help us understand the true delta needed to be concerned with poor response to OMT earlier, and consider interventions – exercise programs, medical therapy, dietary/lifestyle changes, or advanced HF workup – to prevent acute decompensation and urgent need for transplantation or inotrope/LVAD therapy as salvage.

Our data, despite being single center, does highlight a potential area for further opportunity. Understanding the profiles of our patients and stratifying low risk or high risk to progress is increasingly important in our field, underscoring the need that benefits of performing CPET may not be only for patients undergoing transplant assessments.

## 5 Limitations

Our study is thought provoking and hypothesis generating, as are all retrospective assessments. The main limitation is our sample size; however, we remain the largest single center study and at present the only to compare VO_2_ data in the same patient before and after SGLT2i therapy, not only to the OMT or placebo group. As a retrospective study, we were not able to include KCCQ12, 6MW or NT-Pro-BNP assessment, which may provide further details into quality of life in the setting of unchanged CPET studies.

## 6 Conclusions

Our single-center data shows that functional testing in advanced HF patients is not significantly impacted by SGLT2i as assessed by the peak VO_2_ through serial CPET testing. Given the pluripotent nature of the effects of SGLT2i, volume status and reduction in intracardiac filling pressures may improve symptoms and reduce hospitalizations but this does not impact long term likelihood of needing an advanced therapy evaluation. Further studies are needed to fully evaluate the effects of SGLT2i in HF particularly with respect to the standardization of study endpoints and the inclusion of late-stage HF (NYHA class 3+) patients.

## Data Availability

The raw data supporting the conclusions of this article will be made available by the authors, without undue reservation.
